# The Hospital. Nursing Section

**Published:** 1904-07-16

**Authors:** 


### The Hospital

**Nursing Section.**

Contributions for this Section of "The Hospital" should be addressed to the Editor, "The Hospital" Nursing Section, 28 & 29 Southampton Street, Strand, London, W.C.

No. 929.—Vol. XXXVI. *SATURDAY, JULY 16, 1904.*

### Notes on News from the Nursing World.

### RECEPTION OF PENSION FUND NURSES BY HER MAJESTY AT BUCKINGHAM PALACE.

We announced in a special late supplement last week with great pleasure—which we are sure will be fully shared by our readers—that her Majesty, Queen Alexandra, has been graciously pleased to consent to present certificates at Buckingham Palace on Friday, July 22nd, at 3 P.M., to those members of the Royal National Pension Fund for Nurses, who have joined since her Majesty's last reception on July 19th, 1901. On five former occasions the Queen, in her capacity as President of the Fund, invited the nurses to Marlborough House, but this will be the first time that nurses have been received at Buckingham Palace, and should thus become historical. It will naturally be the ardent desire of every nurse who is entitled to a certificate to avail herself of the privilege which the Queen wishes her to enjoy, and it may be assumed that nothing except indisposition or absence from England will prevent any of the members from being present at such a memorable gathering. The rush of nurses from the provinces to London next week will, therefore, be very considerable, and in order to provide for their comfort we ask matrons and superintendents of nursing homes, or others who can accommodate them on reasonable terms on the evening of Thursday, July 21st, to communicate at once with the Secretary of the Pension Fund, 28 Finsbury Pavement, E.C. The Secretary asks us to state that all policy-holders will be communicated with as soon as final details are known; that the preliminary meeting will be held about one o'clock; that the ceremony will be over about 5 P.M.; that the railway companies have agreed to issue return tickets at single fares from stations outside the metropolitan area—10 miles—available from July 20th to the 25th; that cloak-room accommodation, to a limited extent, will be provided at Buckingham Palace; that the nurses must remain on the ground between the preliminary meeting and the termination of the ceremony; and that no nurse can attend the ceremony unless the card sent out by the office has been returned filled in and signed, and the policy number given. The offices of the National Pension Fund will be closed on the day of the reception.

### THE NEW QUEEN'S NURSES.

It will be seen from the official list of New Queen's Nurses, which appears in another column, that 84 have been added to those who serve in England, 16 in Wales, 20 in Scotland, and 12 in Ireland. This is an increase of 13 as compared with the number appointed at the beginning of the present year. As on the last occasion, nine of the new nurses in England received their training in Bloomsbury; of the remainder eight were trained in Liverpool and five each in Manchester, Brighton, and Hull; of those in Wales five were trained in Cardiff. The majority appointed to Scotland were trained in Edinburgh, and the majority to Ireland in Dublin.

### ARMY NURSES AT DINNER.

The annual dinner of Queen Alexandra's Imperial Military Nursing Service was held at St. Andrew's Club, Mortimer Street, a few days ago. Viscountess Downe presided, and there was a large number of members of the old Army Nursing Service and Queen Alexandra's Imperial Military Nursing Service. The guests included Miss Monk, matron of King's College Hospital, and Miss Cave, matron of Westminster Hospital. The health of the King and the President of the Service, Queen Alexandra, was proposed by Lady Downe and drunk with enthusiasm. Lady Downe placed before the members the importance of the work in which Queen Alexandra's Imperial Military Nursing Service is engaged, and exhorted the service to maintain a high standard of excellence. Miss Monk, matron of King's College Hospital, proposed the health of Miss Sidney Browne, the matron-in-chief, and also coupled with the toast the name of Miss Becher, principal matron. She expressed the satisfaction which the civil nursing profession felt in the development of nursing in the Army, and cordially congratulated the members of the service on the steady progress which had been made. Miss Browne returned thanks in a few suitable words. After dinner, by the kindness of Miss E. Debenham, the club-rooms were used by the guests, Miss Debenham and her sister also contributing to the pleasure of the guests by playing some selections on the violin, her sister accompanying her on the piano. Mrs. Freyer and Miss Saunder sang some delightful songs.

### THE FALLACY OF LADY FRANCES BALFOUR.

Lady Frances Balfour, in a letter on Mrs. Alfred Lyttelton's play, in which she blames the law of the land for its infringement by West End dressmakers, incidentally makes an amazing statement. In attempting to prove that "all work for bread has its times of stress and strain and its special danger to health," she says: "The average of death among hospital nurses is appallingly early." We are glad to see that Lord Lytton made haste to correct Lady Frances, by means of a citation from the last report of the Royal National Pension Fund for Nurses, with which our readers are familiar. They will remember that Mr. King, the well-known actuary, showed, in the clearest possible manner, that the valuation of the Fund conclusively establishes the fact that nurses, as a body, are long lived—"longer lived, in fact, than women in other walks of life." Seeing that the members of the Pension Fund embrace a very large proportion of hospital nurses, it would be interesting to know where Lady Frances obtained the information on which she has founded a singular and absolutely exploded fallacy.

### A SCHOOL NURSE FOR BRIGHTON.

Thanks largely to the efforts of Dr. Newsholme, the Medical Officer of Health for Brighton, the Education Committee of the Brighton Town Council have decided to appoint a trained nurse to assist the officers and teachers in tracing and isolating infectious disease and in assisting to emphasise the importance of the regularity of school attendance. Dr. Newsholme, in a letter addressed to the Education Committee, which no doubt greatly influenced them in their decision, stated that he would be glad to give a nurse every possible help in organising her work and instituting the necessary medical directions; and he also expressed the opinion that not only would the attendance at school be improved, but that much unnecessary suffering would be abated through the services of a nurse. The appointment, which is to be made as an experiment for a year, does not involve an addition to the staff under the Council, as the nurse will, in effect, fill the vacancy caused by the retirement of one of the attendance officers. Moreover, as his salary was £125, and that of the nurse is to be £85, the cost to the ratepayers will be £40 less. We have not the slightest doubt that at the end of the year the appointment will be renewed without opposition, if, as it may be anticipated, the useful work done in London by the school nurses is emulated by the new official in Brighton.

### THE VACANCY AT LEWISHAM.

The post of matron of Lewisham Union Infirmary is vacant, and in view of the fact that the institution is a recognised training school of considerable importance the terms of the appointment are interesting. The salary offered is £100 per annum, with furnished apartments, rations, and the usual allowances, which are subject to a deduction under the Poor-law Officers' Superannuation Act of 1896. Candidates must be not less than 28 nor more than 45 years of age, and it is essential that they should have been trained for three years in a general hospital or a Poor-law infirmary maintaining a resident medical officer. Complaints are frequently made that the best Poor-law posts are given to nurses trained in general hospitals; but it may be presumed that the Lewisham guardians will be guided in their choice by the personal qualifications and experience only of the applicants.

### LADY HERMIONE BLACKWOOD'S DISTRICT.

At the annual meeting of the Bangor District Nursing Society, over which the Dowager Marchioness of Dufferin presided, a statement that during the seven months Lady Hermione Blackwood was on duty in the Claudeboye District she had received 48 patients and paid nearly 600 visits, elicited applause. As Miss Borlase, the other Queen's nurse of the society, attended 191 patients in the year and paid 4,688 visits, her services also merit recognition, and we are glad to notice that Lady Dufferin warmly praised the work she had done. In consequence of the unremitting attention of Miss Borlase to her duties, the committee have arranged for her to have a long rest this summer, and have secured another Queen's nurse to take her place till October. We suggest that as the Society is in a prosperous condition, with a balance of £74 to their credit, an arrangement should be made for relieving Miss Borlase permanently of a portion of her work.

### THE TRAINING OF PROBATIONERS AT WARRINGTON INFIRMARY.

At the last meeting of the Warrington Guardians a resolution to the following effect was passed: "That on the satisfactory completion of the three years' course of training the probationers shall receive a certificate of competency provided they have passed such examinations as may be prescribed by the Guardians or the Local Government Board." This is the outcome of a movement which has been going on for some time having in view the more efficient general training of probationers at the Poor-law Infirmary. The difficulty in the path is the fact that there is no resident medical officer, but as the House Committee pointed out that other infirmaries similar in character and size to that of Warrington are training their probationers without possessing a medical man resident on the premises, and that the standard of the probationers was good, the nursing more efficient, and the examination results gratifying, it was decided that the objection was not insuperable. There can, of course, be improvements in the system of training without the appointment of a resident medical officer, but the probationers in such schools cannot qualify for the higher posts in the nursing world.

### STREET PROCESSIONS AND NURSING ASSOCIATIONS.

It seems that nursing associations may suffer from the new police regulations prohibiting street processions and collections. On successive occasions the demonstrations of the trades and friendly societies on behalf of the North London Nursing Association have yielded £18, £25, and £35. This year, no demonstration being allowed, the working-men have made a little private collection and have handed to the hon. secretary the sum of £4 12s. 4d. They are also considering whether they cannot by some other method of collection show in a substantial way the warm feeling of gratitude which they entertain to the association. Meanwhile, the association lose at a time when funds are badly needed a substantial amount.

### BRENTFORD UNION INFIRMARY.

The Matron of Brentford Union Infirmary, gives some interesting details in her report of the eight years of the training school. Since July 1st, 1903, four nurses, who had finished their three years' training and received their certificates, left, two giving up nursing, two to take up other appointments. Two others, who completed their training during the year, were appointed sisters at Isleworth. During the year seven probationers were promoted to be staff nurses and eight new probationers have been appointed. There are now in the training school 23 nurses and probationers. The final examination for nurses in their third year was held last month by Dr. Seymour Sharkey, of St. Thomas's Hospital. Six nurses presented themselves for examination and all passed. Dr. Sharkey, in his report, stated that they all answered fairly well and some very well; and that all displayed a considerable amount of knowledge, intelligence, and interest in their work. Dr. Norton and Dr. Silvester subsequently held the usual annual examination for the junior nurses and probationers, and the result was very satisfactory. Massage classes have been held by Sister Brocks, and all the nurses sent up for the examination held by the Incorporated Society of Trained Masseuses passed and obtained the certificate. This is the first year in which special midwifery instruction has been given at Isleworth. Dr. Tooks delivered one course of midwifery lectures to the sisters and senior nurses, and Dr. Silvester two courses of lectures to the midwifery pupils. The four nurses trained in midwifery during the year were all successful in obtaining the certificate of the Obstetrical Society of London.

### WEST HAM NURSES' EXAMINATION.

At the meeting of the West Ham Board of Guardians on Thursday last week, 21 nurses were presented with their training certificates by the chairman on behalf of the Board. In his congratulatory address he remarked that it was evident from the result of the examination that the nurses had worked very hard to obtain such a gratifying report from the examiner and hoped that the efforts put forth by them would be emulated by others who were to follow. The Examiner, Dr. T. H. Openshaw, stated that the examination was one of the most satisfactory conducted by him and that great credit was due to the several officers whose duty it had been to train and prepare the nurses. The examination was the first held at the new infirmary at Whipps Cross, Leytonstone. The names of the successful candidates are :—Nurses Smith, Moore, Vinter, White, Ray, Weeks, Cropper, Vernon, Lawrie, Dorkins, Hill, Henshaw, Dennett, Tate, Garrett, Simpson, Kingston, Cobham, Elliott, Clark, and Hodnett.

### THE AMBULANCE QUESTION.

At a meeting of the Banbridge Guardians it was stated that a nurse had refused to accompany the ambulance to convey a patient to the workhouse on the requisition of the dispensary medical officer of Tandragee. It was ultimately decided to ask the Local Government Board if it was obligatory for the nurses to accompany the ambulance when requested by the guardians, and if not, what course ought to be pursued in case of an emergency. The suggestion was made that if the superintendent nurse had not intervened no difficulty would have arisen. We certainly think that if the nurse who declined to go out with the ambulance acted on instructions from the superintendent, she was not in any way to blame.

### A NURSING ASSOCIATION IN LOW WATER.

The nurse employed by the Raunds Nursing Association has received a month's notice to leave owing to the inability of the committee to obtain sufficient money to carry on the organisation. At present there is a deficiency of nearly £15, and it is calculated that in another two months, at the end of the financial year, the deficit will have increased to £21. But neither this, nor the statement that there is a depression in the staple trade, nor the plea that there is little illness now in the town, justifies the action which it is proposed to take. As the vicar of Raunds, who attended the meeting which was called to discuss the situation said, the suspension of the Association ought not to be contemplated. It is never "an opportune time" to get rid of an agency for the benefit of the sick poor, and if the Raunds people do not reverse the faint-hearted decision arrived at by the majority of the persons who attended the public meeting, they ought to be ashamed of themselves.

### GARDEN PARTY AT MARPLE BRIDGE.

On Saturday, July 2nd, a garden party, in aid of the Marple Bridge District Nursing Association, was held at Ernecroft, Marple Bridge. A large company assembled to view the garden, and to witness the dancing round the may-pole, and afterwards scarf dances, fan dances and reels on the lawn. Twenty-eight children, from four years of age to 12, took part, having been carefully trained for some time previously. The children, who belonged to the district, were prettily dressed in white, with black shoes and stockings, and presented a charming appearance. Tea was provided in the grounds, and the ladies on the committee, assisted by the villagers, supplied cakes and similar refreshments. The weather, which had been very stormy in the morning, accompanied by thunder and heavy rain, cleared up before the entertainment began, and the remainder of the day was very fine and sunny. A charge of 3d. admission, and 6d. for tea, resulted in the sum of £11 5s. 1d. being added to the funds of the Nursing Association. The services of Miss C. M. Walker, the Queen's nurse, who has worked in the district for about six years, are much appreciated, and she has endeared herself to all classes by her tact and sympathy.

### GARDEN FÊTE AT GONALSTON.

A very successful sale of work and garden fête has just been held in the grounds of Gonalston Hall on behalf of the District Nursing Association. The work of the nurse employed by the association is carried on in the villages of Gonalston, Hoveringham, Thurgarton, and Bleasby, and while a brisk business was done at the stalls, there were plenty of entertainments for the numerous visitors, including a tennis tournament, and dancing on the lawn to the strains of a string quartette.

### SOCIAL GATHERING FOR NURSES.

On Friday last a social gathering for nurses was held at Long Ashton, near Bristol, when, in spite of a disappointment caused by the absence of a lady doctor who was to have addressed the meeting on medical electricity, the guests passed a pleasant afternoon. The subject of a lending library for nurses was discussed favourably. The Nurses' Missionary Union was also talked about, and many of the nurses were interested in hearing of this work which has lately been started.

### SHORT ITEMS.

Miss Sarah E. Webb, R.R.C., has resigned her post as matron in Queen Alexandra's Imperial Military Nursing Service.—The King has sanctioned the appointment of Miss Louisa Twining as Lady of Grace of the Order of the Hospital of St. John of Jerusalem in England.—The Convalescent Home in connection with Ancoats Hospital, Manchester, was opened last Saturday by an "At Home," which was attended by a large gathering of friends who expressed themselves delighted with the arrangements.

### The Nursing Outlook.

"From magnanimity, all fear above;

From nobler recompense, above applause,

Which owes to man's short outlook all its charm."

### NATIONAL AND INTERNATIONAL.

The official report of the first quinquennial meeting of the International Council of Nurses, held at Berlin last month, gives cause for thought. It shows that there were present some 30 nurses from Great Britain, of whom only one (Miss Isla Stewart) was matron of a large London hospital, and other delegates held such positions as the superintendent of Sir Patrick Dun's Hospital, the matron of the General Hospital, Birmingham, the matron of Much Wenlock Hospital, the Queen's nurse from Surbiton, and a sister from the Royal South Hants Hospital. The United States had a somewhat similar representation, and Germany supplied a dozen members from their newly-formed Nurses' Association; and there were single delegates from France, Canada, Denmark, Holland, and Sweden. Was this a representative gathering such as deserved the title "international"? Was the British section deserving of the title of "national"?

First, let it be agreed that there is much good in the thought of international gatherings and communication among nurses, and that there is also much to be said in favour of a National Council of Nurses in every civilised country. But it is a misuse of terms to take a small clique of women who are notorious for not representing the feeling of the mass of nurses and let them arrogate to themselves positions and powers to which they have no right whatever. Here in England the progress of the nursing profession as a whole is terribly hampered by the way some few self-advertising persons try to claim every plan brought forward, which plans at once become anathema when these names are connected with them. Why is it that the matrons of the London hospitals stand aloof from these national councils, from schemes for registration, from the Bedford College scheme for higher education of nurses? We all know quite well that it is simply because the same few are always pushed forward as reformers, and that it is therefore these so-called reformers who are the great hinderers of all nursing progress. It is a strange position; there, blocking the way of all joint action, of all national courtesy, of all unity of aim, are those who boast that theirs is a campaign of nursing reform. Such is the difference in the point of view; to themselves these persons are objects of admiration; to those who take a wider outlook they are obstacles to progress.

To a certain extent this stage army has imposed upon the nurses of the United States, but on no one else. They march on and off the scenes, now as this society and now as that, but like all stage armies they merely show themselves—they do not accomplish anything. And the majority of the audience quite understands; it knows that in spite of blare of trumpets it is only the same few persons marching round and round, and that they are quite ineffective; just a few in the far-off gallery may perhaps be taken in; those of us who are nearest recognise the same faces again and again, though perhaps the helmets may have been hastily changed behind the scenes. Weird little stage army! so mighty in its own imagination, so laughable to the lookers-on! But are we going to allow the stage army to pretend to be an International Council of Nurses? Are we going to allow it to be a National Council of Nurses?

The Select Committee on Nurse Registration has already set to work at Westminster, and its outcome will probably be the formation of some nursing council, even if no registration bills are passed; and that council, if formed, should be the real thing, and not the dressed-up booby of a diminutive clique. In the great strife against death and disease, in the great cause of the procuring of health for our city children, in which nurses are now taking so large a part, there must be no narrowness, no spoiling of the cause because of lack of courage, of want of public spirit. If nurses are to work under a council, be governed by a council, it behoves us to see that that council deserves the title "national," is truly representative, and has for its sole object the raising of the status and the training of nurses in order to benefit humanity as a whole.

And this can only be secured by the vast majority of hospital matrons and nurses forsaking their present Laodicean attitude. "I would that ye were either hot or cold"; for the time has come when the temperature of the nursing world in the future has got to be decided, and unless the truth is put clearly and convincingly before the Select Committee we shall awake some morning to find that the nursing cause has dwindled to something as small and petty as these so-called "national" and "international" councils. When it is possible to adopt such high-sounding titles for such feeble societies, it is evident that push and brag have great power if they are left unopposed. And the humble, unselfish nurse, who is expending her life in the service of the suffering, who is given up to good works, who has no thought of the judgment of this world, and no care for its applause, will find herself forbidden to labour at her vocation save under the rule of those who have openly stated that "a nurse's first duty is to Number One." That matrons and nurses should shrink from coming forward aud joining in the disgraceful quarrels of the nursing world is only natural; but it behoves us now to face the personal strife and petty spite, and, for the sake of the most noble work ever vouchsafed to women, to come forward and speak.

### Lectures Upon the Nursing of Mental Diseases.

By ROBERT JONES, M.D.Lond., B.S., F.R.C.S.Eng., M.R.C.P.Lond., Resident Physician and Superintendent of the London County Asylum, Claybury.

### LECTURE XV.

As to (3) physical drill, this consists in the application of certain exercises for the improvement of the bodily health, and it is significant that insanity is almost always associated with impaired physique and with mal-nutrition, for the report of the Lunacy Commissioners to the Lord Chancellor (1903) shows by statistical tables that impairment of health is intimately connected with mental breakdown. No method of treatment in my experience has been more successful in improving the bodily state and in restoring mental vigour than physical drill. It is especially adapted for some cases, such as those who are suffering from melancholia, or insanity of the dull and depressed type, for cases of stupor, for some cases of premature dementia in young persons, and for those who are "run down,"—neurasthenia—who are irritable or who suffer from so-called nervous debility, or in whom there is a marked tendency to the formation of chilblains, whose circulation is feeble and slow, and who suffer even in warm weather from cold feet and hands. It is also a valuable diversion from overwork, and is a most suitable interruption to mental labour. I am of opinion that the neglect of physical training, and the ignorance of its beneficial effects, is responsible for much distress and poverty, for it favours an ailing existence, and disease is a common source of poverty. By weakening men and women they are rendered unfit to meet their various responsibilities. Outdoor physical drill would do much to counteract intemperance and the bad effects of unhealthy trades and overcrowding. It certainly has done much in asylums to restore that harmony of mind and body which characterises mental health. The movements encouraged by physical drill are educative in their effect. Patients are taught certain bodily movements at the word of command, and to do this with precision and exactitude increases the influence of the will over the muscles of the body, and it tends to independence and presence of mind. It teaches self-reliance, for the combination of muscular movements brings about a prompt, orderly, and precise result in obedience to instructions. The mind perceives the movements, and is thus taught to control and to govern the body, which to be a good servant must be strong. The movements are based upon a correct knowledge of anatomy and physiology, for their effects can be plainly foreseen and demonstrated. The exercises give suppleness and freedom of movement to the frames of those who otherwise would sit about in the wards in constrained attitudes. Drill is the best antidote to a sedentary life, and it has a marked mental effect, for it restricts morbid fancies and helps to prevent patients dwelling upon their delusions. Apart from the purely physical effects of muscular exercise which helps to give a pleasing and graceful appearance to the body, it adds steadiness and dexterity to the frame; making the body straight and firm, it also lends suppleness to movements, helps the circulation, and improves digestion. It opens out the chest—enlarging the narrow one, and thus strengthening the lungs, and by improving the quality and the circulation of the blood and making the skin and the secretions more active, it keeps up a healthy equilibrium between repair and waste. It soothes and favours sleep and has a definite influence upon morality, which those nurses in attendance upon backward children cannot but observe.

The nurse, in exercising her patients, can use this opportunity if she chooses, to encourage a knowledge of ordinary domestic hygiene and combine this with physical development. It is one of the best results of the application of physical drill that in institutions several patients can be systematically taught at the same time. In this way the egotism characteristic of insanity gives way to altruism and friendship, and the fact that these movements take place among several in a class, encourages sympathy and obedience—both of them most desirable features to be re-awakened in the insane. The method, precision, and order involved in physical drill are the best means to recovery in mental diseases, and the patients and the nurse will find much use, as also some amusement, from their application. These free exercises are based upon the Swedish drill introduced by Ling, and they can be carried out in any place, and without any apparatus or appliance other than a cotton or silk sash, which is held stretched out in the hands. The exercises consist in movements of the head, trunk, and limbs, both (in position or) on the spot, as also from the spot with and without support. The arms are raised, the head and neck flexed and rotated, and the legs slide or glide, or are exercised in leaping. These exercises are carried out with regard to time either slowly or quickly, and they may be associated with sound and rhythm such as singing or music. There are five fundamental positions: (i) standing upright with the heels close together and the feet at right angles (ii.) the same with the feet close together and rising on heels and toes; (iii.) striding position with the feet sliding laterally; (iv.) the feet sliding forward or backward as in walking; and (v.) stretching with the arms vertically upwards. These movements are further compounded with flexion and extension of the trunk and limbs, bending forwards or backwards, running, walking, and leaping, and the compounded movements are further elaborated into æsthetic positions and exercises and they are carried out simultaneously with breathing exercises. It is astonishing how dormant paths seem to be re-awakened, ease, certainty, and precision of muscular movements being obtained in the most unpromising and slovenly; all the faculties are re-awakened uniformly and equally, mental and bodily states being harmoniously united into that condition which we call health. I consider physical drill to be a most valuable addition to the treatment of the insane.

As to (4) hydro-therapeutics the term is used to describe the medicinal value of water when applied externally to the body. The use of water is either as a tonic or as a sedative. The tonic effects *i.e.* the general feeling of comfort and the capacity for work, are well known to "cold-tubbers," and the stimulating effects of the cold plunge, of shower baths, and of various kinds of douches are familiar, the reaction, that is the power which the organism has of defending itself against the effects of cold, being different in different individuals. Sea baths or artificial salt baths prepared by the addition of rock salt—any quantity up to 10 lbs. to 30 gallons of ordinary fresh water are also stimulating and a reaction should always be encouraged by vigorous rubbing when cold water either in the form of a spray or a douche is used. Alkaline baths of 30 gallons containing 6 oz. of carbonate of sodium or half this amount of carbonate of potassium and acid baths with 12 oz. of diluted nitro-muriatic acid may be used either hot or cold.

The cold plunge is a momentary stimulation followed by a glowing reaction. A modified application of cold water is by means of the cold sitz-bath. The temperature of the water is that of the surrounding air and the person jumps into the water and out again almost immediately. The shower or rain-bath consists in allowing the water to fall over the head and body from a height and this is borne for about one minute, friction of the body being vigourously applied afterwards. When cold and hot water are used alternately the term Scotch douche has been applied to the process. When the water is directed forcibly to the back of the patient it is called a Charcot douche. These are methods of applying cold water as a tonic, but there is one form which is considered to be "restraint" and notice of the use of this, which is the cold pack, must be sent to the Lunacy Commissioners in the case of persons under a certificate. The method of applying the cold pack is to wring a sheet out of cold water, wrap it about the patient for a few minutes when it is removed and the patient placed comfortably in bed and rubbed to obtain a reaction. Great care must always be taken in giving cold baths to the insane. It is best to begin with warm water and gradually lower the temperature and care must be taken to examine the heart, as cold baths are contra-indicated when the circulation is weak or the patient is elderly, for the stimulation may be in excess of the powers of the organism to react. Cold baths are excellent remedies for hysterical females or young adults suffering from adolescent mania. The bath should not be given against the will of the patient, and all struggling must be avoided. Aromatic and pine-baths, hot or cold, are used for hysterical or nervous patients. They are prepared by adding the essence of pine oil or a decoction of aromatic herbs to an ordinary bath.

As a sedative the various forms of hot or warm baths are used, and their preparation should be familiar to the nurse. The sedative effect is brought about through the healthy reaction of the body to heat, and is shown by a diminished desire for exertion, probably caused by anæmia of the brain and internal organs, brought about by dilatation of the superficial vessels of the skin. The hot wet pack has already been described, and the precautions necessary in its use have been fully referred to for the certified insane. The head is left out of the wet sheet in the wet pack, also often the feet, and the sheet is carried between the legs and wrapped smoothly about the patient, who remains in the pack from half an hour to an hour. Hot water bottles are applied to the feet and cold compresses to the head, but the greatest care is needed in the use of the wet pack, as dangerous collapse may follow its use for excitement. When taken out the patient is sponged with warm water and placed in bed between warm blankets.

The hot sitz-bath is used at a temperature of 100° to 110° for about half an hour, and is a valuable sedative for uterine irritation, hysteria, or neuralgia, complaints not infrequent among patients in both public and private asylums. The continuous warm bath is less used than formerly in the treatment of acute mania. The bath is prepared at 98·4° or 100° and the patient immersed in it. His head is kept above the bath by a boarded covering, there being a hole through which the neck and head protrude. The patient eats, sleeps, and passes all his emunctories in the bath and is continuously immersed for 18 to 24 or 48 hours. In some asylums on the Continent immersion has been continuous for a period of six weeks. The effects of the continuous bath are probably in part a mechanical action caused by the water on the blood-vessels of the skin, as well as probably also a vaso-motor one. The Turkish bath is the application of air to the surface of the body at a temperature of 150° and over. The soaping, rubbing, douching, and cold spray afterwards are a powerful and agreeable stimulation to the skin. The Russian bath is heat applied by vapour and the temperature is necessarily much lower than the hot dry air of a Turkish bath. These various methods of hot water application are excellent remedies for insomnia.

The (5) rest treatment was described and initiated by Weir-Mitchell, and is of especial value in cases of hysteria, neurasthenia and incipient insanity. Its essential features are: (*a*) isolation from the patient's family and from former surroundings; (*b*) rest in bed—"a man's life has often been saved by breaking his leg"—an overworked, overstrained man or woman is kept absolutely and compulsorily in the recumbent position, not even being allowed to sit up in bed; (*c*) massage as a form of gentle exercise or (*d*) electricity as a stimulant being applied, by (*e*) a tactful, bright, and sympathetic nurse whose personal influence is an important factor, and lastly (*f*) the diet is carefully and strictly supervised. Feeding is most important, and that form called "high feeding" is observed, the meals being especially plentiful and frequent in order to withdraw blood into the abdomen by means of the food.

The nurse should administer at least six meals a day, which is greatly in excess of what is customary during health, the excess being in the form of fat, eggs, cream, and the like. To assist the nurse a schedule for the day is drawn up, and may be as follows:—

| | |
|---|---|
| 7 A.M. | Cocoa, cool sponge bath, with rough and vigorous rubbing. |
| 8 A.M. | Breakfast, mostly milk; rest for one hour. |
| 10 A.M. | Eight ounces peptonised food. |
| 11 A.M. | Massage or gentle shampooing applied to the head and neck. |
| NOON. | Milk or soup. |
| 1.30 P.M. | Dinner; rest for one hour. |
| 3.30 P.M. | Eight ounces peptonised milk. |
| 4 P.M. | Electricity (generally faradisation—called the coil-current or the alternating current). |
| 6 P.M. | Supper with milk, preferably oatmeal or some farinaceous preparations. |
| 9 P.M. | Massage and warm sponging, or a warm bath and careful rubbing. |
| Further, | 8 oz. of malt extract to be taken with meals, and a tonic after meals; 8 oz. of peptonised milk with biscuit at bedtime, and a glass of milk during the night. |

This treatment is carried out by the nurse or nurses for a period of about six weeks, and the qualified mental nurse is expected to be familiar with the principles upon which the treatment is based.

### TRAVEL NOTES AND QUERIES.

By our Travel Correspondent.

Holidays in Ilfracombe, etc. (Mrs. L.). If you would like a small house at Brixham with attendance at very reasonable terms send me a stamped and addressed envelope, and I will forward address. Accommodation for three, terms 30s. per week. This sum covers attendance, kitchen fire, and lights. House free after August 1st.

Rules in Regard to Correspondence for this Section.—All questioners must use a pseudonym for publication, but the communication must also bear the writer's own name and address as well, which will be regarded as confidential. All such communications to be addressed "Travel Editor, 'Nursing Section of *The Hospital*,' 28 Southampton Street, Strand." No charge will be made for inserting and answering questions in the inquiry column, and all will be answered in rotation as space permits. If an answer by letter is required, a stamped and addressed envelope must be enclosed, together with 2s. 6d., which fee will be devoted to the objects of "The Hospital" Convalescent Fund. Any inquiries reaching the office after Monday cannot be answered in "The Hospital" of the current week.

### Queen Victoria's Jubilee Institute for Nurses.

HER MAJESTY QUEEN ALEXANDRA has been graciously pleased to approve the appointment of the following as "Queen's Nurses" to date July 1st, 1904:—

### ENGLAND.

| Nurse. | District Training at | Working at |
|---|---|---|
| Minnie Thompson .. .. | Ashton-under Lyne | Ashton-under-Lyne |
| Sarah Ekins .. .. .. | Battersea .. | Tunbridge Wells |
| Agnes Jackman.. .. .. | Bermondsey .. | Doncaster |
| Jane Palmenter.. .. .. | " .. | St. Ives, Hunts. |
| Edith Garratt-Jones .. .. | Blackburn .. | Blackburn |
| Florence Green .. .. .. | " .. | " |
| Sarah Ellen Griffiths .. .. | " .. | " |
| Alice Mary Evans .. .. | Bloomsbury .. | Llantarnam |
| Jean Innes Fortune .. .. | " .. | Willenhall |
| Florence Mary George.. .. | " .. | Bingley |
| Mary Louise Lock .. .. | " .. | Aston-under-Lyne |
| Mildred Charlotte Milman .. | " .. | Bloomsbury |
| Elsie Mary Moss .. .. | " .. | Grantham |
| Martha Reeve .. .. .. | " .. | Matlock |
| Winifred Amy Smith .. .. | " .. | Bloomsbury |
| Jessie Ethel Starling .. .. | " .. | Gloucester |
| Pauline du Moulin Dockeray .. | Bolton .. | Bolton |
| Kate Berkeley-Smith .. .. | Brighton .. | Brixton |
| Minna Clara Delvalle .. .. | " .. | Brighton |
| Hannah Mary Giddins.. .. | " .. | Sandwich |
| Evelyn Maude Greenwood .. | " .. | Hull |
| Ethel Blanche Relton .. .. | " .. | Brighton |
| Hannah Francis Bradford .. | Camberwell .. | Brixton |
| Amy Burkin .. .. .. | Camberwell .. | Blofield |
| Priscilla Jenkins .. .. | " | Camberwell |
| Ethel Cecilia Henderson .. | Cardiff .. | Cleckheaton |
| Florence Worthington.. .. | " | Barrow-in-Furness |
| Blanche McQueen .. .. | Chelsea .. | St. Helen's |
| Gertrude Amelia Swift .. | " | Chelsea |
| Jessie Heriot .. .. .. | Derby C.N.A. | Ashbourne |
| Louisa Rattray .. .. .. | " | Alfreton |
| Ellen Jopson .. .. .. | East London.. | East London |
| Caroline Florence Loweth .. | Gateshead .. | Gateshead |
| Catherine Dyne .. .. .. | " | " |
| Alice Annie Deacon .. .. | Gloucester .. | Wisbech |
| Catherine Higginson .. .. | " | Gloucester |
| Helen Moore .. .. .. | " | " |
| Ida Florence Welch .. .. | Hammersmith | Bramley |
| Eliza Abel .. .. .. | Hull.. .. | Hull |
| Mary Margaret Dorman .. | " | " |
| Martha Knox Mearns .. .. | " | " |
| Annie Milne .. .. .. | " | " |
| Elizabeth Jane Sutton .. .. | " | " |
| Maria Elisabeth Janssen .. | Kensington .. | Kensington |
| Rhoda Elizabeth Harper .. | Leeds (Lovell Street) | Leeds |
| Alice Mary Hughes .. .. | Liverpool (Central) | Liverpool |
| Annie Josephine Bremner .. | Liverpool (Kirkdale Rd.) | " |
| Sarah Ellen Bridgwood .. | " | " |
| Catherine Margaret Bullock .. | " | Blackburn |
| Mary Morgan .. .. .. | Liverpool (Overton St.) | Liverpool |
| Imelda Harriet Tegarty .. | " | " |
| Mary Hughes .. .. .. | Liverpool (Shaw Street) | " |
| Jessie Louisa Prestidge .. | " | Crook |
| Charlotte Eliza Almond .. | Manchester (Ardwick Green) | Manchester |
| Harriet Atkinson .. .. | Manchester (Bradford) | Manchester |
| Kate Teresa Hastings .. .. | Manchester (Harpurhey) | " |
| Catherine Paddon Phillips .. | " | " |
| Alice Elizabeth Pyne .. .. | " | " |
| Honoria Gittins .. .. .. | Manchester (Hulme) | " |
| Ernestine Eardley .. .. | Northampton | Northampton |
| Katharine Lunn .. .. | " | " |
| Gertrude Jane Stansfeld .. | " | " |
| Rhoda Mary Griggs .. .. | Paddington .. | Peterborough |
| Henrietta Ethel Hawkins .. | " | Paddington |
| Mary Catherine Jones .. .. | Portsmouth .. | Hampstead |
| Alice Roberts .. .. .. | " | Gloucester |
| Emily Collins .. .. .. | Reading .. | Reading |
| Elizabeth Miranda Lathlean .. | Ryde .. .. | Ryde |
| Alice Maud Brown .. .. | St. Helen's .. | Hackney |
| Ada Emily Arnold .. .. | Salford .. | Salford |
| Alice Ena Holmes .. .. | " | " |
| Jane Elizabeth Shelton .. | " | " |
| Louisa Anne Young .. .. | " | " |
| Grace Angus .. .. .. | Shoreditch .. | Shoreditch |
| Elizabeth Jane Clague .. .. | " | Ramsbury |
| Lily Ethel Nazer .. .. | " | Hereford |

| Nurse. | District Training at | Working at |
|---|---|---|
| Fanny Newman.. .. .. | Shoreditch .. | Shoreditch |
| Constance Eva Nicholl .. | " | Rochdale |
| Sarah Isabella Bustard .. | Somerset, C. N. A. | Street |
| Charlotte Helen B. Brooks .. | Southampton | Southampton |
| Helen Maria Moxon .. .. | " | Eton |
| Eliza Ann Fisher .. .. | Torquay .. | Torquay |
| Ettie Maria Worthington .. | Walworth .. | Burnham |
| Ida May Purkis .. .. .. | Westminster .. | Gosport |

### WALES.

| Nurse. | District Training at | Working at |
|---|---|---|
| Margaret Templeton .. .. | Barry .. .. | Barry |
| Emily Ada Wisbey .. .. | " | " |
| Katharine Aitkin .. .. | Bermondsey .. | Gowerton |
| Mary Jane Monypeny .. | Brighton .. | Swansea |
| Elizabeth Anne Evans.. .. | Cardiff .. | Milford Haven |
| Cecil Georgina Hanbury .. | " | Cardiff |
| Adelaide Elizabeth Haw .. | " | Ruthin |
| Catherine Jones.. .. .. | " | Broughton |
| Charlotte Elizabeth Lindsay .. | " | Abergele |
| Lilian Heggie .. .. .. | Liverpool (Central) | Penmaenmawr |
| Margaret Arnold James .. | " | Montgomery |
| Constance Agnes Saltmarsh .. | Salford .. | Llanidloes |
| Rose Timms .. .. .. | " | Neath |
| Frances Ethel Morrison .. | Shoreditch .. | Bangor |
| Jeanie Owen .. .. .. | " | Penrhyn Deudraeth |
| Winifred Harris .. .. | Walworth .. | Newtown |

### SCOTLAND.

| Nurse. | District Training at | Working at |
|---|---|---|
| Alice Caroline Andrews .. | Edinburgh .. | Edinburgh |
| Jean Hamilton C. Bain .. | " | Dundee |
| Catherine Black .. .. | " | Edinburgh |
| Mary Drysdale Carstairs .. | " | " |
| Elizabeth Gibson .. .. | " | " |
| Agnes Brodie Gilfillan.. .. | " | " |
| Georgina Gillies .. .. | " | " |
| Catherine Hardisty .. .. | " | " |
| Agnes Kemp .. .. .. | " | Balerno, N.B. |
| Margaret MacArthur .. .. | " | Craignish (Argyleshire) |
| Rebecca McClelland .. .. | " | Dunbar |
| Mary Macdonald Miller .. | " | Carluke |
| Janet Riddock .. .. .. | " | Uddingston |
| Grace Annie Souter .. .. | Edinburgh .. | Aberdeen |
| Sophia Webb .. .. .. | " | Clydesdale |
| Ada Bell Weir .. .. .. | " | Paisley |
| Mary Smith .. .. .. | Dundee .. | Dundee |
| Mary Lindsay Barclay .. | Glasgow .. | Glasgow |
| Margaret Beatson .. .. | " | " |
| Jeanie Falconer Maxwell .. | " | " |

### IRELAND.

| Nurse. | District Training at | Working at |
|---|---|---|
| Kathleen Dobbins .. .. | Clarina .. | Clarina |
| Effie Kate Barr-Hamilton .. | Portsmouth .. | St Lawrence's, Dublin |
| Bridget Kennelly .. .. | St. Lawrence's, Dublin | Dublin |
| Annie Josephine Killeen .. | " | " |
| Annie Leyden .. .. .. | " | Cashel |
| Letitia E. M. Macmahon .. | " | Arranmore |
| Georgina MacWilliam .. .. | " | Achill Island |
| Nellie O'Donnell .. .. | " | Dublin |
| Olga Brauer .. .. .. | St. Patrick's, Dublin | " |
| Mary Waller A. Gillmor .. | " | " |
| Ethel Rutter .. .. .. | " | " |
| Laura Marie Tyner .. .. | " | Arklow |

### Presentations.

STAFFORDSHIRE INSTITUTION FOR NURSES, STOKE-UPON-TRENT.—On Wednesday in last week 74 nurses of the Staffordshire Institution for Nurses, Stoke-on-Trent, assembled at the Home and presented their lady superintendent, Miss Birdsall Hobkinson, who is shortly to be married, with two entrée dishes (inscribed with name and date) and a case of solid silver salts, pepperettes, and mustard pot. Nurse Morris, the senior nurse, who made the presentation on behalf of her fellow nurses, in a few well-chosen words said how sorry they all felt to lose their matron, and wished her every happiness in her married life. Miss Hobkinson, in reply, said that she could not fully express all she felt in thanking them and was really sorry to leave them. A photograph of the lady superintendent and the nurses was taken in the garden, and afterwards they all had tea in the Home before going back to their cases.

### The Registration of Nurses.

### EVIDENCE BEFORE THE SELECT COMMITTEE.

The Select Committee of the House of Commons appointed to consider the expediency of providing for the Registration of Nurses held their first meeting for the purpose of hearing evidence on Thursday last week, Mr. H. J. Tennant presiding.

### Dr. Bedford Fenwick.

The first person to volunteer evidence was Dr. Bedford Fenwick, of Upper Wimpole Street, who said that at present there was no uniformity in the training of nurses. Certificates of efficiency were granted to nurses by hospitals of every size and kind, but the public had no means of discriminating between good and bad, efficient and inefficient nurses. Neither was there any control or discipline over them. Unless a nurse possessed technical knowledge she would be unable to do two things which were most essential for the welfare of her patient. First, she would be unable to carry out with efficiency and knowledge the duties entrusted to her by the doctor; and, secondly, she would be unable to report to the doctor what particular symptoms the patient had shown during his absence. A large number of those who were at present following the profession of nursing were unable to take the temperature and pulse properly. He would not have separate classes of medical, surgical, and obstetric nurses. Every nurse should have a general knowledge of these three branches and she might specialise afterwards on that basis. There were over 80,000 women who were engaged in nursing in the United Kingdom, and, so far as he could make out, there were at least 40,000 of these who could not have had any adequate training. It was essential that Parliament should pass legislation in order to secure some system of uniform training and to prevent the great danger that now proceeded from the want of control over nurses. He was in favour of the Bill introduced by Dr. Farquharson, one of the main provisions of which was the setting up of a central nursing council, which would hold examinations and grant certificates and exercise disciplinary powers over registered nurses. The General Medical Council, in 1889, unanimously resolved that it would be to the advantage of the public, and to the convenience of practitioners, that facilities, under proper guarantees, should be given by Act of Parliament or otherwise for the authoritative certification of trained nurses, and that when certified they should be subject to common rules of discipline. The British Medical Association, in 1896, also unanimously resolved that an Act of Parliament should be passed as soon as possible for providing for the registration and education of medical, surgical, and obstetric nurses. He had no reason to suppose that either of these bodies had, in the interval, changed its views on the subject. He did not think there was any fear of opposition from the nurses to examination and registration, because it would raise the status of the profession, which was now suffering from the entrance of a large number of people unqualified for the work. Nor would it exclude any but well-to-do women from the profession. It would, on the other hand, get rid of many of the evils of the present system.

### The Matron of St. Bartholomew's.

Miss Isla Stewart, Matron of St. Bartholomew's Hospital, gave evidence on Tuesday.

The Chairman, who had a *précis* of Miss Stewart's evidence before him, after some preliminary inquiries as to her position, previous appointments, and training, asked the witness for her views as to wherein the present training of nurses was, as she alleged, deficient.

Miss Stewart replied that, while there was an absolute lack of uniformity in training, small hospitals gave certificates as well as large. In her own case, after six years' training at St. Thomas's Hospital, she had received no certificate at all, and she handed to the Chairman a receipt for £2, which was all she had to show in evidence of the time spent there.

In reply to questions Miss Stewart described fully the course of training and granting of certificates in force at St. Bartholomew's, emphasising her belief that the discipline, both in wards and in the Nursing Home, extending over three years, formed a better preparation for after life than could be obtained in any shorter period.

The Chairman supposed it might be taken for granted that the witness was of opinion that some system of registration, such as had been suggested in the two Bills introduced into the House of Commons this year, would supply the deficiency in uniformity, and that some sort of general council should, in her opinion, be created to examine nurses and grant certificates, to which there was an affirmative reply. She did not anticipate insurmountable difficulties in administration, nor, in reply to a question put by Sir John Batty Tuke, did she see why the proposed examination should become a matter of "grind" and routine. The variation in methods of training in the great hospitals was slight, and she did not fear great divergence of ideas, supposing they became the authorities. It would, she thought, be possible to have an examination general enough to test each individual.

With regard to the question of moral fitness, Miss Stewart acknowledged that the hospital which trained the nurse must be the judge. The advantage to be gained, she believed, by the establishment of an independent examining body, was that it would be proved that all the hospitals were training their nurses sufficiently and efficiently.

Miss Stewart then detailed a scheme of examination, and in reply to Sir John Batty Tuke, defined what she meant by the terms anatomy and physiology as applied to the training of nurses.

Being cross-questioned, she admitted that the training in some small hospitals was efficient, but she was not, apparently, ready with a reply to Sir J. B. Tuke's question, as to what difference it would make where a nurse was trained, so long as she could pass her examination.

### Probationers of Eighteen.

One of the committee was anxious to know which hospital had the severest examination, but on this point Miss Stewart was unable to throw light. She said she would not, however, wish to raise the proposed examination to a greater degree of severity than at present obtained in a good hospital. The question of age being mentioned, Miss Stewart expressed the opinion that 21, or even 20, was not too young for probationers to enter for general training; being pressed, she saw no reason why they should not enter at 18, although admitting that it was rather young. The effect on the public was next dealt with, Miss Stewart maintaining that at present there was a risk of imperfectly trained women being obtained; cases had, indeed, come within her own range of experience, of women dismissed from the hospital for incompetency and general unfitness, who were competing with the fully-trained private nurse.

The Chairman, quoting the argument against registration, summed up in the words "once registered always registered," asked whether the public would be lulled into a false sense of security, to which the witness replied that a registered nurse would always be competent, since her three years' training would be assured. She would make it penal for a woman to exercise the function of a nurse on pretence of having been registered. The proposed Council should in no way interfere with existing training, but should act as an additional safeguard to the nurse and to the public. Cottage nurses, and nurses under the Poor Law, she was understood to say, would be excluded from the working of the scheme. A member of the Committee asked whether anything like a special degree at Cambridge was contemplated; Miss Stewart thought not. Registration would not diminish the supply of nurses, but rather the reverse. It might, however, have the effect of reducing cottage hospitals and cottage nurses. With regard to finance, she thought that a fee of, say, two to three guineas, might be imposed; she believed that nurses would gladly pay this in order to assure their status.

Miss Huxley.

The evidence of Miss Margaret Huxley was next taken. In reply to the Chairman, she said that she had a surgical home in Dublin, and had been 18 years at Sir Patrick Dun's Hospital, having received her training at St. Bartholomew's Hospital. Her evidence was chiefly corroborative of that given by Miss Stewart. She would make 21 years of age the minimum for examination. She did not consider cottage nurses as nurses. She estimated the wastage in the nursing ranks at about 2,000 per annum.

Dr. Bedford Fenwick, being asked for his financial scheme of the proposed council, gave some figures based on the accounts of the General Medical Council.

The two cases, Sir J. B. Tuke said, were not parallel. He could see no relation between the two bodies.

The Chairman said the question of finance would be gone into later, and the committee rose after sitting for two hours.

### Everybody's Opinion.

MALE NURSES AND STATE REGISTRATION.

"Certificated Mental Nurse" writes: I read with interest in your columns a letter written by a certificated male nurse on the "State Registration of Male Nurses," and also your comment upon his letter. I think that the impression of Mr. Bloomfield's remarks on the best type of male nurse would have been more favourable if he had dwelt more on the bright side of his case, as it cannot now be doubted that there is such a side to look upon. He says, "At present the male nurse is looked down upon as being much inferior to his female colleague." An assertion such as this from a fellow male nurse is very unpalatable to the up-to-date colleague of his own sex, who is smart, intelligent, well-trained, respectably educated and who commands not only the respect but also the gratitude of doctors and well-to-do people throughout the country. I have lately been working together with female nurses who had been trained and had held important posts in some of the largest hospitals, and in justice to the nurses themselves, as well as to their employers, I wish to state that I was not made to feel by either that I, as a male nurse, was considered inferior to the female nurses in any respect. I am convinced that the doctors I have worked for and the families I have lived with have not looked down upon me but have shown me such respect as a female nurse trained in a first-class hospital would have reasonably expected. Nor am I an exception, as I have among my friends quite a number of male nurses who succeed in making a good impression when in attendance on their cases, and I understand that all the best class of male nurses receive the same treatment. Mr. Bloomfield states that the unaccountable prejudice against us as a class is largely owing to the fact that many of these unskilled men who are engaged in nursing and who call themselves trained nurses have not had the opportunity to have an efficient training, and therefore cannot be trusted to undertake grave responsibility such as a nurse often has to take." Well, the female nurses surely vary from the highly-trained nurse of the best training schools to the nurse who has only received a few months' training; and do we not read sometimes of women posing as trained nurses, parading the country in nurse's uniforms, and accepting nurse's fees who have not had a day's training inside a hospital? So that if there are among the male nurses those who are not up to the highest standard of efficiency, the same is true of the opposite sex. No doubt it will be readily acknowledged that the male mental nurses, who have completed their training lately, and those who are at present in training, are much superior to their predecessors, thanks to the efforts of the Medico-Psychological Association. The fact of the Association having instituted an examination and granted certificates for proficiency in mental nursing, and having considerably raised the standard of the training and examination, has induced men of better qualities and education to take up the profession of mental nursing. This brings me to the last point which I wish to deal with, in which I find myself partly in agreement with Mr. Bloomfield, viz., that in order that the male nurse should be given fair play the wards of the general hospitals should be thrown open to him. You seem to consider it doubtful whether the wards of the general hospitals could ever prove attractive to young men of education to enter them as nurses. But the woman is now elbowing the man out at every point, and a large number of educated young men find it very difficult to obtain situations where they can earn salaries equal to those paid to well-trained female nurses. With reference to State registration, cannot the certificates of the Medico-Psychological Association, and those of the National Hospital, serve as passports to registration for the male nurse, as they are the only ones granted by competent authorities?

### Appointments.

Barton-upon-Irwell Union.—Miss Esther A. Thompson has been appointed charge nurse. She was trained at St. Olave's Infirmary, Rotherhithe, London, where she has since been charge nurse. She has also been charge nurse at the Union Workhouse, Bury, Lancashire.

Darlington Fever Hospital.—Miss E. M. Radcliffe has been appointed sister. She was trained at Sunderland Infirmary and at the Cardiff Sanatorium. She has also done private nursing.

Denny and Dunipere Cottage Hospital.—Miss Hortensia Elvira de Cot has been appointed nurse-matron. She was trained at Glasgow Royal Infirmary and has since been senior sister at Bromhill Home, Kirkintilloch, and charge nurse at Johnstone Cottage Hospital.

Holborn Union Schools Infirmary.—Miss Annie T. Hyde has been appointed superintendent nurse. She obtained her ophthalmic experience at Hanwell Ophthalmic School, and was trained at St. Saviour's Infirmary, East Dulwich.

London Orphan Asylum, Watford.—Miss Hannah N. Rutt has been appointed infirmary matron. She was trained at Leicester Infirmary and has since been theatre sister at Lewisham Infirmary; sister at the Great Northern Central Hospital, London; and sister in charge of the Infirmary Infant Orphan Asylum.

Newton Abbot Hospital.—Miss A. W. Houlding has been appointed staff nurse. She was trained at Chorlton Union Infirmary, West Didsbury, Manchester.

Wakefield Union Infirmary.—Miss Kate Stokes has been appointed sister. She was trained at the Middlesbrough Union Infirmary.

West Derby Union Infirmary, Liverpool.—Miss Irene C. Lowndes has been appointed charge nurse. She was trained at the Royal Southern Hospital, Liverpool, and at the Royal Infirmary, Liverpool. She has since been on the private staff of the Royal Infirmary, Liverpool.

West Malling Workhouse Infirmary.—Miss Amy Laird has been appointed assistant nurse. She was trained for two years at St. Peter's Home, Woking, by the Meath Workhouse Nursing Association.

### The Modern Nursing of Consumption.

The United Alkali Company ask us to call attention to the fact that in Lecture IX. of the above series Chloros is mentioned as being obtainable from the Company at Birmingham, whereas the address should have been James Street, Liverpool.

### Echoes from the Outside World.

### Movements of Royalty.

On Saturday the King travelled to Sandringham for the purpose of inspecting the work of reconstruction and redecoration necessitated by the outbreak of fire in the Queen's apartments last December. On the tour of inspection his Majesty was attended by the staffs of the architect and contractor, and they received various expressions of approval and valuable suggestions from the King, who always displays the greatest interest in any building operations upon the estate. After he had lunched at Sandringham his Majesty paid a visit of inspection to Appleton Lodge, which is being enlarged to meet the increasing accommodation needed by Prince and Princess Charles of Denmark.

On Monday afternoon the Prince and Princess of Wales visited the Leysian Mission, City Road, London, to perform the ceremony of opening the Queen Victoria Hall, a handsome building capable of accommodating 2,000 persons. There are 125 other rooms to be used for settlement and other purposes, six complete suites of club-rooms being set apart for the use of the dwellers in the neighbourhood, who have little besides the public-house they can call "home." There are also four "roof-gardens." Lord Strathcona informed the Prince of Wales that the mission was founded in 1886 and since that time had been entirely supported by the past and present scholars of the Leys School, Cambridge, assisted by a devoted staff and voluntary helpers. His Royal Highness in reply said that he was glad that the physical and recreative element entered so considerably into the scheme. Subsequently, the Princess received purses for the fund.

### The War in the Far East.

It is officially reported that the second Japanese army, under General Oku, after dislodging the Russians from their positions, occupied Kai-ping and the neighbouring heights on Saturday. On Friday night, during a storm, a flotilla of torpedo-boats from Admiral Togo's fleet approached Port Arthur. One of the boats discovered the Russian cruiser *Askold* on Saturday morning and attacked her. Four Japanese torpedo-boats made an attack on Port Arthur on Sunday but were repulsed without loss. On July 7th there was severe fighting round Port Arthur. The Russians claim to have driven the Japanese back, but admit having lost over 1,000. On Sunday the Czar, after reviewing the 5th and 6th army corps under orders for the Far East, said he was confident that they would uphold the honour of the Russian arms and wished them God-speed, both in his own name and that of the Czarina.

### The Alake's Departure.

The Alake of Abeokuta left Liverpool on Saturday for Lagos, and was accorded a hearty send-off by a large crowd, which assembled on the landing-stage. Shortly before his departure from London, the King sent the Alake a present of a Bible, with a gracious letter, expressing the hope that he had enjoyed his visit, and wishing him a safe journey home. Inside the Bible was an inscription, stating that it had been presented by King Edward the Seventh to the Alake of Abeokuta, July 1904, to replace the Bible given by Queen Victoria in 1848 to Sagbua, father of the present Alake, which was lost in a fire 20 years after. The Bible was handed over to the sole charge of an attendant, who guarded it jealously. The Alake stated that his first act upon his return to Abeokuta would be to hold a thanksgiving service in the church to thank God for all His goodness, for the hearty and warm reception of the English people, and especially for the graciousness of the King. He then proposes to make a tour of his country to see where, and to what extent, he can put into practice some of the things he has learnt with regard to cotton growing; to urge upon his people kindness to animals; to discourage the use of the native bits so hurtful to the horses; and to take steps to make roads such as the Alake saw in England.

### Changes in the Colonial Service.

Several important changes in the Colonial Service have been approved by the King. Sir Gerald Strickland, Governor of the Leeward Islands, becomes Governor of Tasmania, He is a Count of the Holy Roman Empire, a Maltese noble with a palace in Valetta, and a villa at San Antonio. His English home is the castle and fortress of Sizergh in Westmorland. Sir James Swettenham, Governor of British Guiana, has been made Governor-in-Chief of Jamaica and its Dependencies, in succession to Sir Augustus Hemming. Sir Clement Courtenay Knollys, at present Colonial Secretary of Trinidad and Tobago, becomes Governor of the Leeward Islands; and Sir Frederick Hodgson, Governor of Barbados, takes the place of Sir James Swettenham as Governor of British Guiana. Sir F. Hodgson commenced life in the General Post Office, and did not enter the Colonial Service until 1888.

### The Church Council and the Female Franchise.

The Church Council comprising the Houses of Convocations of the two Provinces and Houses of Laymen discussed last week the question of female franchise. Chancellor P. V. Smith moved "That it is desirable that the initial franchise of lay electors should be extended so as not wholly to exclude women, and that the presidents be requested to appoint a committee to consider and report to the Council at their next sitting how this extension should be carried out. The Bishop of Worcester, in seconding the motion, urged that women had always been given positions in the Church, and to exclude them wholly from the franchise would be going back at the very moment of their highest weight of influence. Lord Hugh Cecil in opposing the admission of women to even a portion of the initial franchise, affirmed that the current democratic theories were not applicable to the Church, because they were foreign to the whole conception of Church construction. Ultimately, the resolution was carried, the votes being 153 for it and 58 against. Two dilatory amendments were defeated by substantial majorities.

### Actors' Orphanage Fête.

On Friday the Royal Botanic Gardens were crowded, the occasion being the Actors' Orphanage Fête. Tickets to the number of 7,000 were sold beforehand, and as many more were paid for at the doors. The weather was splendid. The Prince and Princess of Wales were present, and the entertainment was well planned and well executed. The startling, pathetic, and entrancing melodrama entitled "The Track of Blood," written by Captain Robert Marshall, attracted great numbers of visitors, who were invited to "walk up" by Mr. Brandon Thomas, standing on a platform, beating big drums in old-fashioned style. The cricket match between actors (playing left-handed) and actresses was another feature of interest, and caused much fun, although, owing to the heat and limited space, there was no running, the umpires awarding runs according to the strength or direction of each hit. Perhaps the most amusing was the sight of some of London's celebrated actors trimming hats, which they afterwards put on. The Prince and Princess of Wales laughed heartily at the way Mr. George Alexander solemnly threaded his needle and then started work, work which was so successful that he won the first prize. The Æolian Ladies' Orchestra, Zarega mandolines and guitars, "The Follies," the Honourable Artillery Band, and idyllic players in pastoral plays, all added to the enjoyment of the afternoon.

### Notes and Queries.

**FOR REGULATIONS SEE PAGE 143.**

*Text-books.*

(112) Will you be kind enough to recommend me a text-book dealing with the minor operations, especially of women, and one dealing with the different instruments used in ordinary hospital operations (illustrated)?—*A. B. C.*

"Surgical Ward Work," by Dr. Miles, The Scientific Press, 29 Southampton Street, Strand.

*Male Nurse.*

(113) Will you kindly advise me how I can become a male nurse; I should prefer fever and mental work?—*J. C.* and *F. L. B.*

Write first to the National Hospital for the Paralysed and Epileptic, Queen's Square, Bloomsbury, W.C. After two years' training there you can study for either fever or mental nursing.

*Aching Feet.*

(114) I should be much obliged if you will kindly tell me a remedy for aching feet, consequent on much walking in hot weather?—*S. E.*

Bathing the feet in hot water, in which a little washing soda has been dissolved, often affords relief. Wear boots with soft leather, and rest the feet as much as possible. Your local doctor could probably help you.

*Rome.*

(115) Can you tell me of other nursing homes in Rome besides the Anglo-American? I am anxious to do private nursing out there, but should prefer, if possible, to join a private home.—*R. L. P.*

Write to an English medical man or English chaplain. Either might help you.

*Paris.*

(116) Will you kindly give me the name of an institute for English nurses in Paris? Also the address of the English hospital.—*M. E. R.*

There is no institute for English nurses in Paris. The address of the Hertford British Hospital is Rue de Villiers, Levallois-Perret, Paris.

*Workhouse Training.*

(117) After receiving training in a Poor-law infirmary should I be eligible for district nursing, or only for a workhouse appointment?—*D. M. C.*

You would be equally eligible for either if you were trained for three years.

*Male Probationer.*

(118) Will you tell me where I could train, other than the National Hospital, Bloomsbury, and what time of the year is best for vacancies?—*W. E. H.*

There is no hospital, except the one you mention, where male probationers are trained. The asylums and the Royal Army Medical Corps, however, train their own probationers.

I wish to obtain a situation as attendant at an asylum or hospital.—*David L.*

The National Hospital for Paralysis and Epilepsy, Bloomsbury, is the only hospital which trains male probationers. Write to the asylums.

*Training in South Africa.*

(119) Can a lady of 29, wishing to be a nurse, train in South Africa? She meant to train here, but has a chance of going out to South Africa and still wishes to be a nurse.—*Y. O.*

Yes, either at the Johannesburg Hospital or at the Provincial Hospital, Port Elizabeth, Cape Colony. Both have training schools.

*Nurses' Home in Cairo.*

(120) I am desirous of obtaining an address of a nursing institute in Cairo?—*J. T.* (*Edinburgh*).

The nursing homes in Cairo are private, and we do not give private addresses. Write to the English medical man or the English chaplain.

### Handbooks for Nurses.

"How to Become a Nurse: The Nursing Profession." 2s. net; 2s. 4d. post free.

"The Nurses' Dictionary." By Honnor Morten. 2s. post free.

"A Handbook for Nurses." By J. K. Watson, M.D. 5s. net; 5s. 4d. post free.

"A Practical Guide to Surgical Bandaging and Dressings." By W. Johnson Smith, F.R.C.S. 2s. post free.

"The Art of Massage." By A. Creighton Hale. 6s. post free.

"Preparation for Operation in Private Houses." By Stanmore Bishop, F.R.C.S. 6d. post free.

The above works are obtainable of all booksellers, or direct from The Scientific Press, Limited, 28 & 29 Southampton Street, Strand, London, W.C.

### For Reading to the Sick.

### "I AM WITH THEE."

A little child in pain or grief
Runs quickly to his mother's side,
Assured, whatever may betide,
Her sympathy shall bring relief.

She tells him that 'tis not for long,
This strange new sorrow—dries his tears,
Her circling arm allays his fears,
He sees her face, and he is strong.

'Tis thus God comforts those who flee
In childlike trust to His wide breast;
They hear—and hearing, calmly rest—
"Fear not, for I am still with thee."

And as they lift their eyes above,
The thought of His almighty power
Is less their strength in trial's hour
Than the sweet knowledge of His love!

*Harriet E. Colvile.*

No earthly friend may be near; but in every furnace there is One like the Son of Man. In every flood of high waters He stands beside us—staying the heart with promises, instilling words of faith and hope, recalling the blessed past, pointing to the radiant future, hushing fear, as once He stilled the dismay of His disciples on the lake: such is the ministry of Jesus. And as the sufferer looks back on the trial, he says, "I never felt Him so near before; and if it had not been for what He was to me, I could never have lived through it."

There is nothing harder to bear than the apparent aimlessness of sorrow. . . . With the Christian there is no fear of this. There is a utility in every trial. It is intended to reveal the secrets of our heart; to humble us and prove us; to winnow us, as corn is shaken in a sieve; to detach us from the earthly and visible; to create in us an eager desire for the realities which can alone quench our cravings and endure for ever.—*Rev. F. B. Meyer.*

In the dark hours of our life all other sounds die away and leave silence in our souls. Silence that we may hear His voice. And it is a great step forwards in the Christian life if one learns to say, "The Lord is my portion." Nothing teaches this as sorrow teaches. From it we learn the transitoriness of earthly things, the permanence of the eternal, the loving call of God; but also we learn the very hard lesson that God is really the only satisfaction for the soul.

*Mrs. Romanes.*

Deep in the heart of pain God's hand hath set
A hidden rest and bliss;
Take as His gift the pain, the gift brings yet
A truer happiness:
God's voice speaks, through it all, the high behest
That bids His people enter into rest.

*Lucy Fletcher.*

